# Risk factors for acute kidney injury after major abdominal surgery in the elderly aged 75 years and above

**DOI:** 10.1186/s12882-022-02822-7

**Published:** 2022-06-23

**Authors:** Jianghua Shen, Yanqi Chu, Chaodong Wang, Suying Yan

**Affiliations:** 1 Department of Pharmacy, Xuanwu Hospital, Capital Medical University, National Clinical Research Center for Geriatric Diseases, 45 Changchun Street, Beijing, 100053 Xicheng District China; 2 National Clinical Research Center for Geriatric Diseases, Beijing, 100053 China

**Keywords:** Acute kidney injury, Postoperative AKI, Abdominal surgery, Elderly patients, Nephrotoxic drugs, Risk factors

## Abstract

**Objectives:**

The study aimed to investigate the incidence and risk factors of acute kidney injury (AKI) in elderly patients (aged ≥ 75 years) undergoing major nonvascular abdominal surgery.

**Methods:**

The study was a retrospective study that evaluated the incidence of AKI in patients within 48 h after major abdominal surgeries. Patients' preoperative characteristics and intraoperative management, including the use of nephrotoxic medications, were evaluated for associations with AKI using a logistic regression model.

**Results:**

A total of 573 patients were included in our analysis. A total of 33 patients (5.76%) developed AKI, and 30 (90.91%), 2 (6.06%) and 1 (3.03%) reached the AKI stages 1, 2 and 3, respectively. Older age (adjusted OR, aOR 1.112, 95% confidence interval, CI 1.020–1.212), serum albumin (aOR 0.900, 95% CI 0.829–0.977), baseline eGFR (aOR 3.401, 95% CI 1.479–7.820), the intraoperative occurrence of hypotension (aOR 3.509, 95% CI 1.553–7.929), and the use of hydroxyethyl starch in combination with nonsteroidal anti-inflammatory drugs (aOR 3.596, 95% CI 1.559–8.292) or furosemide (aOR 5.724, 95% CI 1.476–22.199) were independent risk factors for postoperative AKI.

**Conclusions:**

Several risk factors, including intraoperative combined administration of HES and furosemide, are independent factors for AKI during abdominal surgeries. Anesthesiologists and surgeons should take precautions in treating at-risk patients.

## Background

Acute kidney injury (AKI) is defined as an abrupt decrease (within hours) in kidney function that encompasses both injury (structural damage) and impairment (loss of function). AKI occurrence complicates the recovery course and worsens the outcome in hospitalized patients [1]. Patients with AKI have a higher risk of developing chronic kidney disease (CKD) and end-stage renal disease, resulting in morbidity, mortality, and increased economic burden [1, 2]. Proper management of AKI offers survival benefits [3]. Identifying risk factors for AKI is critical to developing preventive and therapeutic management strategies for the occurrence of AKI.

Surgery is a major factor for AKI. The literature on perioperative AKI focuses primarily on cardiac and major vascular surgeries. General intra-abdominal surgery has been identified as a high risk for developing AKI among noncardiac general surgery procedures. The prevalence of AKI after abdominal surgery was 0.8% to 22.4% [4–7]. AKI risk factors after abdominal surgery include older age, high body mass index (BMI), male gender, and morbidities of type 2 diabetes, hypertension, and ischemic heart disease [6–8]. Furthermore, the perioperative use of nephrotoxic drugs, such as furosemide, hydroxyethyl starch (HES), non-steroidal anti-inflammatory drugs (NSAIDs), and contrast agents, can negatively influence perioperative renal function and are considered as possible risk factors for postoperative AKI [9–11].

Very few studies investigated risk factors for AKI in elderly patients after abdominal surgery. Therefore, this retrospective cohort study was aimed to identify the incidence and risk factors (clinical characteristics, comorbidities, intraoperative use of nephrotoxic drugs) of postoperative AKI in the first 48 h after major non-vascular abdominal surgery in elderly 75 years and older.

## Methods

### Patients and study design

We conducted a retrospective cohort study of patients aged ≥ 75 years who underwent scheduled major non-vascular abdominal surgery from January 2016 to December 2020 at Xuanwu Hospital, Capital Medical University, China. Major abdominal surgery was defined as whenever the intraperitoneal approach was performed under general anesthesia, and the predictable length of stay for patients in a given diagnosis-related group exceeded two days [3]. Exclusion criteria were 1) End-stage kidney disease (ESKD) patients receiving renal replacement therapy (RRT) or kidney transplant recipients, and 2) patients without at least a baseline serum creatinine (SCr) value and at least two Scr values during hospitalization on two different days. Only the first surgery was considered for analysis for patients undergoing more than one surgery within the same hospitalization.

### Variables and data collection

A data collection sheet was used to collect the demographic (age, gender, body mass index) and clinical characteristics of the relevant patients. The following clinical data were collected 1) preoperative disease states: hypertension, diabetes mellitus, ischemic heart disease, congestive heart failure (CHF), cerebrovascular disease, chronic obstructive pulmonary disease (COPD), solid malignancy, and hematologic malignancy, 2) preoperative laboratory values: hemoglobin, SCr, serum albumin (ALB), alanine aminotransferase (ALT), and aspartate aminotransferase (AST), 3) surgical approach (laparoscopy, laparotomy), 4) surgical site (colorectal, gastric, hepato-biliary-pancreatic, small bowel), 5) intraoperative characteristics: operative time (min), duration of anesthesia (min), intraoperative occurrence of hypotension (IOH), amount of crystalloids use (L), and blood loss (mL), and 6) intraoperative drugs: vasoactive drugs, and nephrotoxic drugs (hydroxyethyl starch HES, NSAIDs, furosemide). The American Society of Anesthesiologists (ASA) score, the Charlson Comorbidity Index (CCI) score, and the Revised Cardiac Risk Index (RCRI) score were calculated and recorded. All variables were collected from electronic clinical records, including intraoperative data recorded by anesthesiologists.

### Definitions and calculations

The Kidney Disease: Improving Global Outcomes (KDIGO) criteria were used to define and stage AKI severities [12]. SCr measurements on the first and second postoperative days were compared with preoperative SCr (baseline) measurements. AKI was defined based on changes in SCr: an increase in SCr by ≥ 0.3 mg/dL (≥ 26.5 µmol/L) within 48 h or an increase in SCr to ≥ 1.5 times baseline. Therefore, AKI was classified into stage 1 (SCr 1.5–1.9 times baseline or ≥ 26.5 µmol/L increase), stage 2 (SCr 2.0–2.9 times baseline), and stage 3 (SCr ≥ 3 times baseline or postoperative SCr ≥ 354 μmol/L with elevations of at least 26.5 μmol/L from baseline or initiation of RRT) [13]. The estimated preoperative glomerular filtration rate (eGFR) was calculated using the CKD-EPI equation. For a more detailed analysis of the cohort according to preexisting kidney function, patients were classified into two groups: the CKD group (eGFR < 60 mL/min/1.73m^2^) and the non-CKD group (eGFR ≥ 60 mL/min/1.73m^2^) [12]. Acute kidney disease (AKD) refers to a prolonged kidney injury (functional and/or structural abnormalities) that continues for at least seven days and maximum of 90 days [12].

Intraoperative systolic blood pressure (SBP) and diastolic blood pressure (DBP) were recorded every 5 min. Mean arterial pressure (MAP) was calculated as (2/3DBP + 1/3SBP). When blood pressure was measured both invasively and noninvasively, invasive measurements were used for analysis. IOH was defined as MAP < 65 mmHg [14]. The ASA score was used to evaluate the preoperative physical state based on five classes (I to V) [15]. The RCRI score was calculated to identify patients at risk of developing postoperative complications. Each risk factor is assigned a single point: high-risk surgical procedure, history of ischemic heart disease, history of CHF, history of cerebrovascular disease, preoperative treatment with insulin, and preoperative Scr > 2.0 mg/dL [16]. The intraperitoneal procedure was considered high risk in all cases in this study.

### Statistical analysis

Continuous variables are expressed as mean ± standard deviation and categorical variables as the number and percentage of cases. Comparisons between patients with and without AKI were performed using the Student’s *t*-test or the Mann–Whitney U test for numerical data and χ2 test or Fisher’s exact test for categorical data. Independent predictors of AKI were evaluated with the logistic regression method. The risk factors for AKI were first evaluated with univariate analysis, and the statistically significant variables *P* < 0.05 were included in the multivariate analysis with forward conditional elimination of data. Data are presented as odds ratios (OR) with 95% confidence intervals (CI). The Hosmer–Lemeshow test was used to test the goodness of fit for logistic regression models. A two-tailed *P* value < 0.05 was considered significant. Analysis was performed using SPSS 23.0.

## Results

**Fig. 1:**
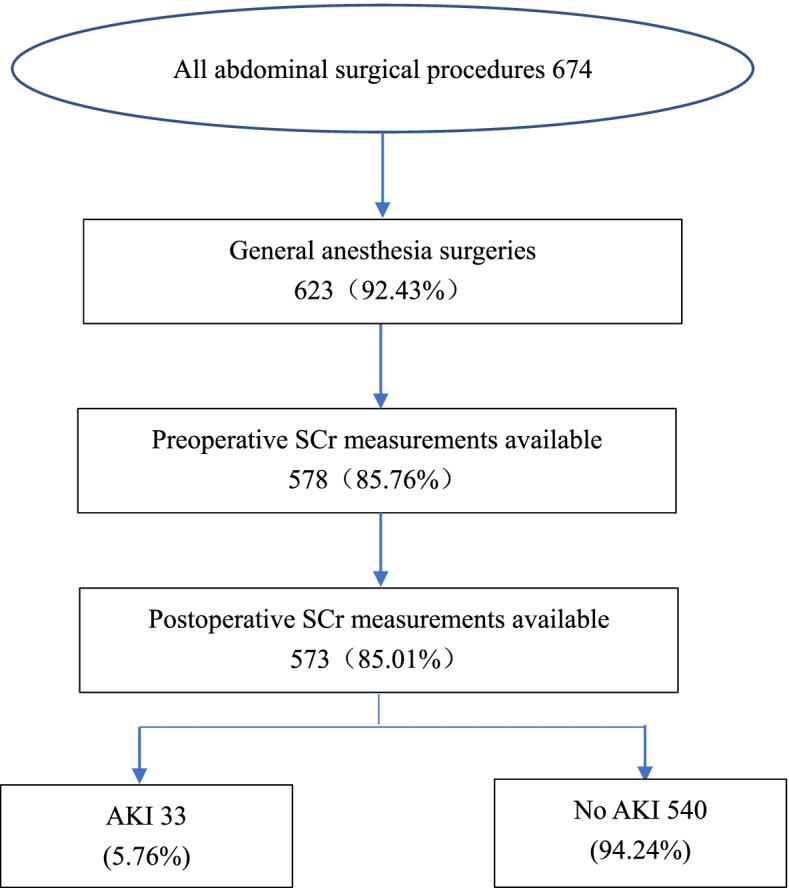
The flow chart of the patient selection. AKI = acute kidney injury; SCr = serum creatinine

There were 674 patients initially included in this study, of whom 623 underwent general anesthesia surgery, and 50 patients were excluded due to missing SCr values (8.03%). A total of 573 patients met the inclusion and exclusion criteria and were included in the analysis (Fig. 1). Not all patients had their SCr values measured on the first and second postoperative days: 12 patients (2.09%) had missing SCr values on the first postoperative day, and 67 (11.69%) had absent SCr values on the second postoperative day. Among these patients, 33 (5.76%) developed AKI, with thirty patients (90.91%) classified as stage 1, two patients (6.06%) as stage 2 and one patient (3.03%) as stage 3. There were 139 laparoscopic and 434 laparotomy surgeries, and the incidences of AKI were 11 (7.91%) and 22 (5.07%), respectively (Table 1).

**Table 1:** Characteristics of patient, medication, and procedure-related factors in patients with and without AKI after abdominal surgery

Factors	All patients (n = 573)	AKI (*n* = 33; 5.76%)	non-AKI (*n* = 540; 94.24%)	*P*
**Preoperative clinical characteristics**				
Male, n(%)	297(51.83%)	11 (33.33%)	286 (52.96%)	*0.032*
Age ± SD, years	81.30 ± 4.433	83.12 ± 4.574	80.66 ± 4.109	*0.001*
Body mass index ± SD(kg/m^2^)	23.50 ± 3.608	23.13 ± 3.806	23.53 ± 3.601	0.537
Hypertension, n(%)	359(62.65%)	19 (57.58%)	340 (62.96%)	0.535
Diabetes mellitus, n(%)	131(22.86%)	9 (27.27%)	122 (22.59%)	0.535
Ischemic heart diseases, n(%)	128(22.34%)	8 (24.24%)	120 (22.22%)	0.787
Congestive heart failure, n(%)	20(3.49%)	2 (6.06%)	18 (3.33%)	0.734
Cerebrovascular diseases, n(%)	98(17.10%)	5 (15.15%)	93 (17.22%)	0.759
COPD, n(%)	12(2.09%)	2 (6.06%)	10 (1.85%)	0.311
Solid malignancy, n(%)	234(40.84%)	11 (33.33%)	223 (41.30%)	0.366
Hematological malignancy, n(%)	2(0.35%)	0	2 (0.37%)	1.000
**Preoperative laboratory test**				
Serum hemoglobin (g/L)	119.59 ± 18.549	115.44 ± 21.437	119.84 ± 18.372	0.193
Serum albumin (g/L)	35.32 ± 4.707	32.12 ± 4.742	35.51 ± 4.643	< *0.001*
Alanine aminotransferase (u/L)	39.07 ± 75.260	73.45 ± 86.665	36.95 ± 74.142	*0.024*
Aspartate aminotransferase(u/L)	44.75 ± 86.239	83.45 ± 106.515	42.36 ± 84.451	*0.037*
Baseline eGFR (mL/min/1.73 m^2^)	70.66 ± 26.686	63.69 ± 23.753	95.47 ± 381.386	*0.633*
Baseline eGFR (mL/min/1.73 m^2^) < 60	76(13.26%)	15(45.45%)	61(11.30%)	< *0.001*
ASA score	2.89 ± .522	3.18 ± 0.635	2.87 ± 0.510	*0.001*
ASA score > 3, n(%)	45(7.85%)	8(24.24%)	37(6.85%)	< *0.001*
Charlson score	6.31 ± .023	7.15 ± 2.694	6.26 ± 1.969	0.071
Charlson score > 7, n(%)	222(38.74%)	15 (45.45%)	207 (38.33%)	0.415
RCRI score	0.53 ± 0.672	0.85 ± 0.755	0.51 ± 0.663	*0.005*
Non-renal RCRI score	0.52 ± 0.665	0.76 ± 0.751	0.50 ± 0.657	*0.033*
**Surgical approach**				0.213
Laparoscopic, n(%)	139(24.26%)	11 (7.91%)	128 (92.09%)	
Laparotomy, n(%)	434(75.74%)	22 (5.07%)	412 (94.93%)	
**Operative site**				0.817
Colorectal, n(%)	151(26.35%)	8 (24.24%)	143 (26.48%)	
Gastric, n(%)	30(5.24%)	3 (9.09%)	27 (5%)	
Hepato-biliary-pancreatic, n(%)	274(47.82%)	15 (45.45%)	259 (47.96%)	
Small bowel, n(%)	118(20.59%)	7 (21.21%)	111 (20.56%)	
**Intraoperative characteristics**				
Duration of anesthesia (min)	200.28 ± 113.031	202.97 ± 81.471	200.12 ± 114.836	0.850
Operative time (min)	142.94 ± 100.231	141.52 ± 77.704	143.03 ± 101.594	0.933
IOH, n(%)	116(20.24%)	16 (48.48%)	100 (18.52%)	< *0.001*
Amount of crystalloids use (L)	337.70 ± 340.428	378.79 ± 395.883	335.19 ± 337.324	0.476
Blood loss (mL)	68.52 ± 162.091	48.45 ± 5.242	69.74 ± 166.503	0.465
Vasoactive drug use, n(%)	472(82.37%)	26 (78.79%)	446 (82.59%)	0.578
Usage of nephrotoxic drugs				
Hydroxyethyl starch, n(%)	210(36.65%)	5 (15.15%)	205 (37.96%)	*0.008*
NSAID, n(%)	75(13.09%)	2 (6.06%)	73 (13.52%)	0.333
Hydroxyethyl starch + NSAID, n(%)	101(17.63%)	12 (36.36%)	89 (16.48%)	*0.004*
Hydroxyethyl starch + furosemide, n(%)	13(2.27%)	5 (15.15%)	8 (1.48%)	< *0.001*

*AKI* acute kidney injury, *COPD* Chronic obstructive pulmonary disease, *eGFR* estimated glomerular filtration rate, *ASA* American Society of Anesthesiologists, *RCRI* Revised Cardiac Risk Index, *IOH* intraoperative occurrence of hypotension, *NSAIDs* nonsteroidal anti-inflammatory drugs

Data were presented as median (interquartile range) for continuous variables or number(percentage) for categorical variables

In the 33 patients with AKI, 26 patients (78.79%) had normalized SCr values within a week. One patient (3.03%) required RRT on postoperative day 2 based on the deterioration of renal function and clinical status. The patient Scr returned to normal on the postoperative day 14, and RRT treatment was discontinued. Four patients (12.12%) were admitted to the intensive care unit (ICU) 3–7 days after surgery due to postoperative complications but ultimately recovered within 13–22 days and were discharged. Two patients (6.06%) died on postoperative days 31 and 74 due to sepsis after surgery. Seven patients had abnormal Scr values for more than seven days and met the diagnostic criteria for AKD. Mortality or ICU admission data was not collected among the non-AKI patients, therefore, whether AKI associated with ICU admission or increased mortality was not analyzed.

Demographic, clinical characteristics and perioperative factors in patients with or without AKI were compared (Table 1). Patients who developed AKI were older (83.12 *vs*. 80.66 years, *P* = 0.001) with more females (66.67% *vs*. 47.04%, *P* = 0.032), higher ASA score (3.18 *vs.* 2.87, *P* = 0.001), to be in ASA IV/V (24.24% *vs.* 6.85%, *P* < 0.001), and had a higher RCRI score (*P* = 0.005) and non-renal RCRI score (*P* = 0.033). There were no significant differences in comorbidities between the two groups. Among preoperative laboratory variables, ALB values were significantly lower (32.12 *vs.* 35.51 g/L, *P* < 0.001), and the ALT (*P* = 0.024) and AST values (*P* = 0.037) were significantly higher in the AKI group than in the non-AKI groups. More patients with eGFR < 60 mL/min/1.73 m^2^ developed postoperative AKI (45.45% *vs.* 11.3%, *P* < 0.001). Furthermore, patients with AKI were more likely to have IOH (*P* < 0.001), intraoperative use of HES (*P* = 0.008), and combined use of HES with NSAIDs (*P* = 0.004) and HES with furosemide (*P* < 0.001).

**Fig. 2:**
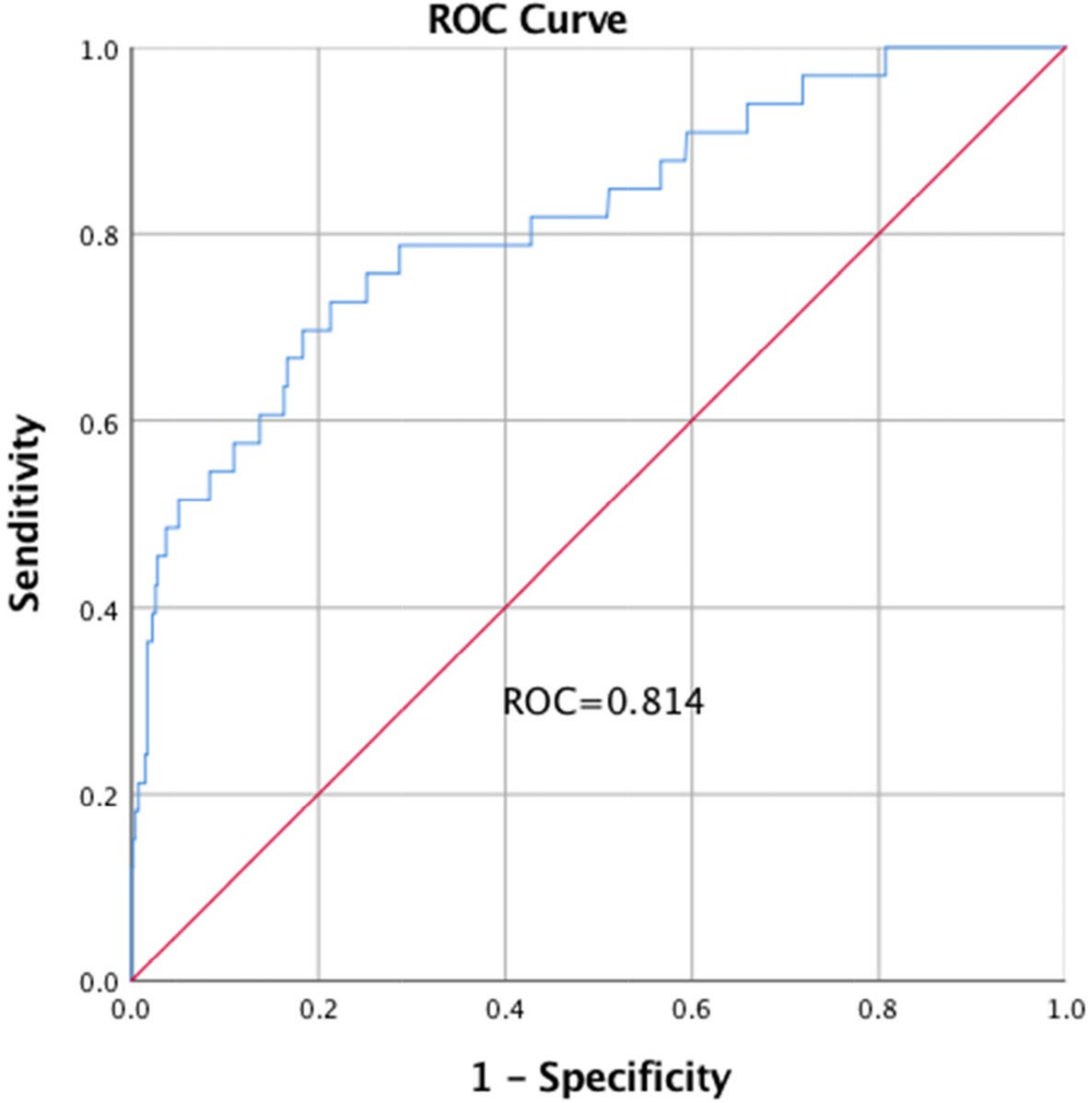
Area under receiver operating characteristic curve for postoperative AKI

We performed a multivariate logistic regression analysis to determine the preoperative and intraoperative risk factors for the development of AKI. Variables included age, sex, preoperative ALB, ALT, and AST, baseline eGFR < 60, ASA score, RCRI, non-renal RCRI scores, IOH, HES, HES with NSAIDs, and HES with furosemide. We identified that age (adjusted OR, aOR = 1.112; 95% CI, 1.020–1.212), serum albumin (aOR = 0.900; 95% CI, 0.829–0.977), eGFR < 60 (aOR = 3.401; 95% CI, 1.479–7.820), IOH (aOR = 3.509; 95% CI, 1.553–7.929) and combined therapy of HES with NSAIDs (aOR = 3.596; 95% CI, 1.559–8.292) or HES with furosemide (aOR = 5.724; 95% CI, 1.476–22.199) were independent predictors of the development of postoperative AKI (Table 2). The Hosmer–Lemeshow test for multivariate models revealed good fits (*χ*^2^ = 4.119, *P* = 0.846). The AUROC was 0.814 (Fig. 2).

**Table 2:** Univariate and multivariate analysis to determine risk factors of postoperative AKI after abdominal surgery

	Univariate analysis		Multivariate analysis	
	Unadjusted OR (95% CI)	*P*	Adjusted OR (95% CI)	*P*
Male	0.444 (0.211–0.934)	*0.032*		
Ages	1.129 (1.048–1.216)	*0.001*	1.112 (1.020–1.212)	*0.016*
Body mass index	0.969 (0.878–1.070)	0.537		
Hypertension	0.798 (0.392–1.627)	0.535		
Diabetes mellitus	0.535 (0.582–2.837)	0.535		
Ischemic heart diseases	1.120 (0.493–2.547)	0.787		
Congestive heart diseases	1.871 (0.415–8.428)	0.734		
Cerebrovascular diseases	0.858 (0.323–2.281)	0.759		
COPD	3.419 (0.718–16.285)	0.311		
Solid malignancy	0.711 (0.338–1.495)	0.366		
Serum hemoglobin	0.988 (0.969–1.006)	0.193		
Serum albumin	0.863 (0.802–0.929)	< *0.001*	0.900 (0.829–0.977)	*0.012*
Alanine aminotransferase	1.004 (1.001–1.007)	*0.024*		
Aspartate aminotransferase	1.003 (1.000–1.005)	*0.037*		
Baseline eGFR (mL/min/1.73 m^2^) < 60	6.544 (3.137–13.649)	< *0.001*	3.401 (1.479–7.820)	*0.004*
ASA score	3.063 (1.584–5.923)	*0.001*		
Charlson score	1.203 (1.035–1.399)	*0.071*		
RCRI score	1.895 (1.204–2.981)	*0.005*		
Non-renal RCRI score	1.654 (1.034–2.645)	*0.033*		
**Surgical approach(**Laparoscopic**)**	0.623 (0.294–1.319)	0.213		
**Intraoperative characteristics**				
Duration of anesthesia	1.000 (0.997–1.003)	0.850		
Operative time	1.000 (0.996–1.003)	0.933		
IOH	4.141 (2.023–8.477)	< *0.001*	3.509 (1.553–7.929)	*0.003*
Amount of crystalloids use	1.000 (0.999–1.001)	0.476		
Blood loss	0.999 (0.995–1.002)	0.465		
Vasoactive drug use	0.783 (0.330–1.857)	0.578		
Usage of nephrotoxic drugs				
Hydroxyethyl starch	0.292 (0.111–0.768)	*0.008*		
NSAID	0.413 (0.097–1.761)	0.333		
Hydroxyethyl starch + NSAID	2.896 (1.375–6.098)	*0.004*	3.596 (1.559–8.292)	*0.003*
Hydroxyethyl starch + furosemide	11.875 (3.648–38.654)	< *0.001*	5.724 (1.476–22.199)	*0.012*

*COPD* Chronic obstructive pulmonary disease, *eGFR* estimated glomerular filtration rate, *ASA* American Society of Anesthesiologists, *RCRI* Revised Cardiac Risk Index, *IOH* intraoperative occurrence of hypotension, *NSAIDs* nonsteroidal anti-inflammatory drugs, *OR* odds ratio, *CI* confidence interval

## Discussion

Postoperative AKI remains one of the leading causes of mortality, prolonged hospital stay, and increased hospital costs [6–8, 17]. In our analysis, about 8% of patients did not have an available SCr value before and after surgery. Romagnoli et al. [18] demonstrated that 14% of patients undergoing a scheduled major abdominal surgery did not have SCr values available. Efforts to obtain SCr values should be made before and after scheduled surgeries to assess patient’s kidney function and to prevent the occurrence of kidney injury, especially in elderly patients.

Our study reveals that the incidence of AKI was 5.76% in the elderly aged 75 years and older who underwent major nonvascular abdominal surgery, and 3.03% of the patients required RRT. Li et al. [4] and Kheterpal et al. [19] reported overall AKI rates of 1.1% and 1.0% after intra-abdominal surgeries. In our study, the incidence of AKI was lower than in several other studies. Causey et al. [20] reported an incidence of 11.8% in patients subjected to colorectal surgeries. Chen et al. [21] reported an AKI rate of 10.9% in elderly patients with chronic hypertension undergoing major gastrointestinal surgery. Teixeira et al. [6] found an incidence of AKI of 22.4% after abdominal surgeries. Direct comparison of these studies is difficult, as AKI criteria, age restrictions, surgical types, and hospital settings are inconsistent. In our study, we were unable to collect data on urinary output. Therefore, the assessment of AKI according to SCr alone could not have identified AKI in patients with low urine output. Additionally, some patients did not have Scr values before and after surgeries, and this could also be the reason for the low incidence of AKI. Many studies confirmed that increased mortality and incident CKD risks are associated with AKD [22, 23]. Although no relevant data were collected in this study, it can be intuitively seen that the prognosis of the seven AKD patients was poor (four admitted to ICU, two died, and one required RRT).

The key in the management of AKI is prevention, as there are no effective pharmacotherapies for AKI. Various predictive models have been developed to stratify risk in patients undergoing surgeries. In our study, after controlling for confounders, age, baseline ALB, eGFR, IOH, and combined treatment of HES with NSAIDs or HES with furosemide were independent predictors of the development of postoperative AKI. Age is a well-known risk factor for renal function impairment in studies [5, 8, 24, 25]. The capacity of the kidney to adapt to hemodynamic changes decreases with age, and even minor injuries can produce functional impairment. Impaired renal function, lower eGFR [4, 7, 26, 27], and ALB [28, 29] are also known factors for postoperative AKI during perioperative surgeries. Patients should be screened for these risk factors before surgery. CKD is associated with increased perioperative morbidity and mortality, even when adjusted for other variables such as hypertension or diabetes [30], and preoperative eGFR should be evaluated in all elderly patients. During the perioperative period, close monitoring of renal function should be provided to patients with preexisting renal insufficiency to prevent the occurrence of AKI.

Similar to other studies, we also found an association between IOH episodes and postoperative AKI [31, 32]. An ischemic insult during intraoperative hypotension could explain why IOH can predict the risk of AKI. Unlike studies that demonstrated an RCRI score and some complications (hypertension and solid malignancy) as risk factors for the development of postoperative AKI [6, 25, 28, 33], we did not find this association.

Large multi-center, non-blinded randomized control trials and meta-analyses have raised concerns about the safety of HES solutions in terms of adverse renal events and mortality [29, 34, 35]. Most of these studies were cardiac and vascular procedures and some included urological procedures that could affect kidney function. The intraoperative use of HES was associated with AKI in patients undergoing major abdominal surgery in our study. After assessing the data submitted by the companies and scientific literature, the European Medicines Agency Pharmacovigilance Risk Assessment Committee (PRAC) suggested that patients treated with HES were at greater risk of kidney injury than those treated with crystalloids [27, 36]. Many studies reported that diuretics could also increase AKI risk [5, 28, 34, 37], especially loop diuretics [38]. The degree of renal injuries was positively correlated with the dose of the diuretic [39–42]. Research has shown that the combined use of diuretics and other nephrotoxic agents could lead to renal dysfunction more than using diuretics alone [43]. Therefore, the recent KDIGO guidelines do not recommend using loop diuretics to prevent or treat AKI [13]. In our study, the combination therapy of HES with NSAIDs or furosemide was an independent risk factor for AKI occurrence. The result is consistent with Landoni et al., study [44]. Anesthesiologists should avoid using HES combined with nephrotoxic drugs as intraoperative medications to reduce the risk of postoperative AKI in elderly patients.

The strengths of our study were the selected study population, patients aged 75 years and older, and using multivariate analysis to identify risk factors. Our study has the following limitations: 1) the study results were from a single hospital, and the results may not be generalized to other settings, 2) the study was a retrospective review of the chart with a small sample size, 3) urine output was not available to assess the occurrence of AKI, 4) not all patients had SCr values on the first and second postoperative days, which explained the lower incidence of AKI in this study compared to other studies, and 5) the long-term consequence of post-surgical AKI was not studied.

## Conclusions

AKI after abdominal surgery is a common occurrence in elderly patients. Several risk factors, especially HES combined with other nephrotoxic drugs, are associated with the development of AKI. Surgeons and anesthesiologists must be vigilant when treating at-risk patients during the perioperative period.

## Data Availability

The datasets generated and/or analysed during the current study are not publicly available due to Ethics Committee requirement of confidentiality of patients' key information, but are available from the corresponding author on reasonable request.

## Abbreviations

AKI: Acute kidney injury
CKD: Chronic kidney disease
BMI: Body mass index
HES: Hydroxyethyl starch
NSAIDs: Nonsteroidal anti-inflammatory drugs
ESKD: End-stage kidney disease
RRT: Renal replacement therapy
SCr: Serum creatinine
CHF: Congestive heart failure
COPD: Chronic obstructive pulmonary disease
ALB: Serum albumin
ALT: Alanine aminotransferase
AST: Aspartate aminotransferase
IOH: Intraoperative occurrence of hypotension
ASA: American Society of Anesthesiologists
CCI: Charlson Comorbidity Index
RCRI: Revised Cardiac Risk Index
KDIGO: Kidney Disease: Improving Global Outcomes
eGFR: Estimated glomerular filtration rate
AKD: Acute kidney disease
SBP: Intraoperative systolic pressure
DBP: Diastolic blood pressure
MAP: Mean arterial pressure
OR: Odds ratio
CI: Confidence interval
ICU: Intensive care unit
PRAC: Pharmacovigilance Risk Assessment Committee

## References

[1] Petaja L, Vaara S, Liuhanen S, Suojaranta-Ylinen R, Mildh L, Nisula S, Korhonen AM, Kaukonen KM, Salmenpera M, Pettila V (2017). Acute Kidney Injury After Cardiac Surgery by Complete KDIGO Criteria Predicts Increased Mortality. J Cardiothorac Vasc Anesth.

[2] Hobson C, Ozrazgat-Baslanti T, Kuxhausen A, Thottakkara P, Efron PA, Moore FA, Moldawer LL, Segal MS, Bihorac A (2015). Cost and Mortality Associated With Postoperative Acute Kidney Injury. Ann Surg.

[3] Zhang J, Feng G, Yang Y, Zhang P, Pu C, Zhao G (2014). Acute kidney injury after radical gastrectomy: a single center study. Int Urol Nephrol.

[4] Kim M, Brady JE, Li G (2014). Variations in the risk of acute kidney injury across intraabdominal surgery procedures. Anesth Analg.

[5] Kheterpal S, Tremper KK, Englesbe MJ, O'Reilly M, Shanks AM, Fetterman DM, Rosenberg AL, Swartz RD (2007). Predictors of postoperative acute renal failure after noncardiac surgery in patients with previously normal renal function. Anesthesiology.

[6] Teixeira C, Rosa R, Rodrigues N, Mendes I, Peixoto L, Dias S, Melo MJ, Pereira M, Bicha Castelo H, Lopes JA (2014). Acute kidney injury after major abdominal surgery: a retrospective cohort analysis. Crit Care Res Pract.

[7] Long TE, Helgason D, Helgadottir S, Palsson R, Gudbjartsson T, Sigurdsson GH, Indridason OS, Sigurdsson MI (2016). Acute Kidney Injury After Abdominal Surgery: Incidence, Risk Factors, and Outcome. Anesth Analg.

[8] Romagnoli S, Zagli G, Tuccinardi G, Tofani L, Chelazzi C, Villa G, Cianchi F, Coratti A, De Gaudio AR, Ricci Z (2016). Postoperative acute kidney injury in high-risk patients undergoing major abdominal surgery. J Crit Care.

[9] Chronopoulos A, Cruz DN, Ronco C (2010). Hospital-acquired acute kidney injury in the elderly. Nat Rev Nephrol.

[10] Degoul S, Chazard E, Lamer A, Lebuffe G, Duhamel A, Tavernier B (2020). Intraoperative administration of 6% hydroxyethyl starch 130/0.4 is not associated with acute kidney injury in elective non-cardiac surgery: a sequential and propensity-matched analysis. Anaesth Crit Care Pa.

[11] Gameiro J, Fonseca JA, Neves M, Jorge S, Lopes JA (2018). Acute kidney injury in major abdominal surgery: incidence, risk factors, pathogenesis and outcomes. Ann Intensive Care.

[12] Lameire NH, Levin A, Kellum JA, Cheung M, Jadoul M, Winkelmayer WC, Stevens PE, Conference P (2021). Harmonizing acute and chronic kidney disease definition and classification: report of a Kidney Disease: Improving Global Outcomes (KDIGO) Consensus Conference. Kidney Int.

[13] Kidney Disease: Improving Global Outcomes (KDIGO) Acute Kidney Injury Work Group (2012). KDIGO clinical practice guideline for acute kidney injury. Kidney Inter Suppl.

[14] Sun LY, Wijeysundera DN, Tait GA, Beattie WS (2015). Association of intraoperative hypotension with acute kidney injury after elective noncardiac surgery. Anesthesiology.

[15] American Society of Anesthesiologists (1963). New classification of physical status. Anesthesiology.

[16] Lee TH, Marcantonio ER, Mangione CM, Thomas EJ, Polanczyk CA, Cook EF, Sugarbaker DJ, Donaldson MC, Poss R, Ho KK (1999). Derivation and prospective validation of a simple index for prediction of cardiac risk of major noncardiac surgery. Circulation.

[17] Yu J, Park HK, Kwon HJ, Lee J, Hwang JH, Kim HY, Kim YK (2018). Risk factors for acute kidney injury after percutaneous nephrolithotomy: Implications of intraoperative hypotension. Medicine (Baltimore).

[18] Villa G, De Rosa S, Calabrisotto CS, Nerini A, Saitta T, Degl'Innocenti D, Paparella L, Bocciero V, Allinovi M, De Gaudio AR et al: Perioperative use of serum creatinine and postoperative acute kidney injury: a single-centre, observational retrospective study to explore physicians' perception and practice. Perioper Med-London 2021, 10(1). 10.1186/s13741-021-00184-6 PMC8145835 34030728

[19] Kheterpal S, Tremper KK, Heung M, Rosenberg AL, Englesbe M, Shanks AM, Campbell DA (2009). Development and validation of an acute kidney injury risk index for patients undergoing general surgery: results from a national data set. Anesthesiology.

[20] Causey MW, Maykel JA, Hatch Q, Miller S, Steele SR (2011). Identifying risk factors for renal failure and myocardial infarction following colorectal surgery. J Surg Res.

[21] Wu XJ, Jiang ZM, Ying J, Han YY, Chen ZH (2017). Optimal blood pressure decreases acute kidney injury after gastrointestinal surgery in elderly hypertensive patients: A randomized study Optimal blood pressure reduces acute kidney injury. J Clin Anesth.

[22] James MT, Levey AS, Tonelli M, Tan Z, Barry R, Pannu N, Ravani P, Klarenbach S, Manns BJ, Hemmelgarn BR: Incidence and Prognosis of Acute Kidney Diseases and Disorders Using an Integrated Approach to Laboratory Measurements in a Universal Health Care System. Jama Netw Open 2019, 2(4). 10.1001/jamanetworkopen.2019.1795 PMC6450331 30951162

[23] Xiao YQ, Cheng W, Wu X, Yan P, Feng LX, Zhang NY, Li XW, Duan XJ, Wang HS, Peng JC et al: Novel risk models to predict acute kidney disease and its outcomes in a Chinese hospitalized population with acute kidney injury. Sci Rep-Uk 2020, 10(1). 10.1038/s41598-020-72651-x PMC7519048 32973230

[24] Cho E, Kim SC, Kim MG, Jo SK, Cho WY, Kim HK (2014). The incidence and risk factors of acute kidney injury after hepatobiliary surgery: a prospective observational study. BMC Nephrol.

[25] Vaught AJ, Ozrazgat-Baslanti T, Javed A, Morgan L, Hobson CE, Bihorac A (2015). Acute kidney injury in major gynaecological surgery: an observational study. BJOG.

[26] Masoomi H, Carmichael JC, Dolich M, Mills S, Ketana N, Pigazzi A, Stamos MJ (2012). Predictive factors of acute renal failure in colon and rectal surgery. Am Surg.

[27] An YB, Shen K, Ye YJ (2018). Risk factors for and the prevention of acute kidney injury after abdominal surgery. Surg Today.

[28] Kim CS, Oak CY, Kim HY, Kang YU, Choi JS, Bae EH, Ma SK, Kweon SS, Kim SW (2013). Incidence, predictive factors, and clinical outcomes of acute kidney injury after gastric surgery for gastric cancer. PLoS ONE.

[29] Lee EH, Kim HR, Baek SH, Kim KM, Chin JH, Choi DK, Kim WJ, Choi IC (2014). Risk Factors of Postoperative Acute Kidney Injury in Patients Undergoing Esophageal Cancer Surgery. J Cardiothor Vasc An.

[30] Eilers H, Liu KD, Gruber A, Niemann CU (2010). Chronic kidney disease: implications for the perioperative period. Minerva Anestesiol.

[31] Salmasi V, Maheshwari K, Yang D, Mascha EJ, Singh A, Sessler DI, Kurz A (2017). Relationship between Intraoperative Hypotension, Defined by Either Reduction from Baseline or Absolute Thresholds, and Acute Kidney and Myocardial Injury after Noncardiac Surgery A Retrospective Cohort Analysis. Anesthesiology.

[32] Walsh M, Devereaux PJ, Garg AX, Kurz A, Turan A, Rodseth RN, Cywinski J, Thabane L, Sessler DI (2013). Relationship between Intraoperative Mean Arterial Pressure and Clinical Outcomes after Noncardiac Surgery: Toward an Empirical Definition of Hypotension. Anesthesiology.

[33] Grams ME, Sang Y, Coresh J, Ballew S, Matsushita K, Molnar MZ, Szabo Z, Kalantar-Zadeh K, Kovesdy CP (2016). Acute Kidney Injury After Major Surgery: A Retrospective Analysis of Veterans Health Administration Data. Am J Kidney Dis.

[34] Schortgen F, Lacherade JC, Bruneel F, Cattaneo I, Hemery F, Lemaire F, Brochard L (2001). Effects of hydroxyethylstarch and gelatin on renal function in severe sepsis: a multicentre randomised study. Lancet.

[35] Winkelmayer WC, Glynn RJ, Levin R, Avorn J (2003). Hydroxyethyl starch and change in renal function in patients undergoing coronary artery bypass graft surgery. Kidney Int.

[36] PRAC: PRAC recommends suspending marketing authorisations for infusion solutions containing hydroxyethyl-starch 2013.

[37] Levi TM, Rocha MS, Almeida DN, Martins RTC, Silva MGC, Santana NCP, Sanjuan IT, Cruz CMS (2012). Furosemide is associated with acute kidney injury in critically ill patients. Braz J Med Biol Res.

[38] Dreischulte T, Morales DR, Bell S, Guthrie B (2015). Combined use of nonsteroidal anti-inflammatory drugs with diuretics and/or renin-angiotensin system inhibitors in the community increases the risk of acute kidney injury. Kidney Int.

[39] Bagshaw SM, Delaney A, Haase M, Ghali WA, Bellomo R, Bellomo R (2007). Loop diuretics in the management of acute renal failure: a systematic review and meta-analysis. Critical Care and Resuscitation.

[40] Ho KM, Sheridan DJ (2006). Meta-analysis of frusemide to prevent or treat acute renal failure. BMJ.

[41] Ho KM, Power BM (2010). Benefits and risks of furosemide in acute kidney injury. Anaesthesia.

[42] Sampath S, Moran JL, Graham PL, Rockliff S, Bersten AD, Abrams KR (2007). The efficacy of loop diuretics in acute renal failure: Assessment using Bayesian evidence synthesis techniques. Crit Care Med.

[43] Wu X, Zhang W, Ren H, Chen X, Xie J, Chen N (2014). Diuretics associated acute kidney injury: clinical and pathological analysis. Ren Fail.

[44] Landoni G, Bove T, Szekely A, Comis M, Rodseth RN, Pasero D, Ponschab M, Mucchetti M, Azzolini ML, Caramelli F (2013). Reducing Mortality in Acute Kidney Injury Patients: Systematic Review and International Web-Based Survey. J Cardiothor Vasc An.

